# Are the Genes *nadA* and *norB* Involved in Formation of Aflatoxin G_1_?

**DOI:** 10.3390/ijms9091717

**Published:** 2008-09-09

**Authors:** Kenneth C. Ehrlich, Leslie L. Scharfenstein, Beverly G. Montalbano, Perng-Kuang Chang

**Affiliations:** Southern Regional Research Center, 1100 Robert E. Lee Blvd, P.O. Box 19687, New Orleans, LA 70179, USA. E-Mails: les.scharfenstein@ars.usda.gov (L.S.); beverly.montalbano@ars.usda.gov (B.M.)

**Keywords:** *Aspergillus parasiticus*, aflatoxin biosynthesis, aryl alcohol dehydrogenase, gene disruption, OYE-flavin mononucleotide reductase

## Abstract

Aflatoxins, the most toxic and carcinogenic family of fungal secondary metabolites, are frequent contaminants of foods intended for human consumption. Previous studies showed that formation of G-group aflatoxins (AFs) from *O-*methylsterigmatocystin (OMST) by certain *Aspergillus* species involves oxidation by the cytochrome P450 monooxygenases, OrdA (AflQ) and CypA (AflU). However, some of the steps in the conversion have not yet been fully defined. Extracts of *Aspergillus parasiticus* disruption mutants of the OYE-FMN binding domain reductase-encoding gene *nadA* (*aflY*) contained a 386 Da AFG_1_ precursor. A compound with this mass was predicted as the product of sequential OrdA and CypA oxidation of OMST. Increased amounts of a 362 Da alcohol, the presumptive product of NadA reduction, accumulate in extracts of fungi with disrupted aryl alcohol dehydrogenase-encoding gene *norB*. These results show that biosynthesis of AFG_1_ involves NadA reduction and NorB oxidation.

## 1. Introduction

**Figure 1.**
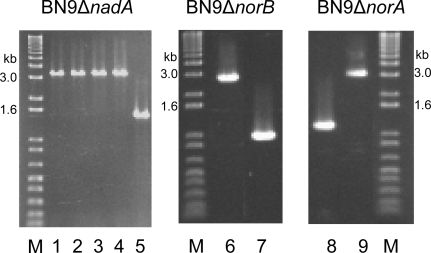
PCR confirmation of BN9Δ*nadA,* BN9Δ*norA,* and BN9Δ*norB* disruptants. Lanes 1–5, PCR of DNA from BN9Δ*nadA* clones 1–5 was with oligos nad1500 and nad2970. Lanes 6, 7, PCR of DNA from BN9Δ*norA* transformant clones A21 and A22 was with oligos norAK and norAH. Lanes 8, 9, PCR of DNA from BN9Δ*norB* transformant clones B4 and B19 was with oligos norBK and norBH. The higher mol wt band in lanes 1–4, 6, and 9 is evidence of insertion of the 2 kb *ptr* gene into the targeted gene.

Aflatoxins (AFs) are *Aspergillus* secondary metabolites that are highly toxic to certain animal species, in particular, birds and fish, and are potent carcinogens [4]. *A. flavus* produces only B-group AFs while *A. parasiticus*, *A. nomius*, and several other species produce both B- and G-group AFs (AFB_1_, AFB_2_, AFG_1_, AFG_2_) [1]. AF biosynthesis requires the expression of at least 28 clustered genes [17]. The roles in biosynthesis of some of the genes have not yet been defined. Although structurally, G-group AFs differ from those of the B-group by having an extra oxygen atom in the A-ring, the conversion requires a number of steps. The initial step in the conversion of *O*-methylsterigmatocystin (OMST) to AFs is oxidation by the cytochrome P450 monooxygenase, OrdA (AflQ) [16]. The G-group AFs are not formed directly by oxidation of B-group AFs but instead require oxidation by the cytochrome P450 monooxygenase, CypA (AflU), of an intermediate common to both types of AFs (Figure 1) [9].

**Scheme 1.**
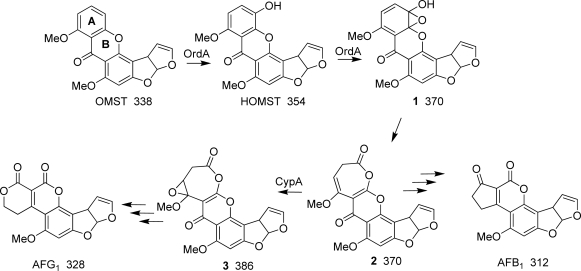
Oxidation by OrdA and CypA in the conversion of OMST to AFB_1_ and AFG_1_. The structures of the 370 Da and 386 Da intermediates have not been proven. Three arrows indicates that multiple steps are involved. Compound numbers in bold font and molecular weights (Da) in normal font are given below the structure

11-HydroxyOMST (HOMST) was proven to be a precursor of AFB_1_ [13] and is the expected product from OrdA-catalyzed oxidization of the A-ring of OMST (Figure 1). Because expression of *ordA* in yeast enabled the yeast to convert OMST to AFB_1_, it was assumed that OrdA also catalyzes the oxidation of HOMST. This oxidation is expected to produce a 370 Da intermediate **1** or **2** (Scheme 1) which was presumed to rearrange non-enzymatically to AFB_1_. CypA oxidation of the 370 Da intermediate would produce a 386 Da AFG_1_ precursor, **3**.

In *A. flavus*, portions of *cypA* and the neighboring gene, *norB* (*aflF*), which encodes a putative aryl alcohol dehydrogenase, are deleted [7]. Loss of the ability to produce G-group AFs in *A. flavus* is due to the *cypA* mutation [7]. The role of *norB* in AF biosynthesis is unknown. Whether or not oxidation by CypA is sufficient to produce G-group AFs is unclear since the substrate for CypA has not been isolated. Formation of AFG_1_ following cytochrome P450-catalyzed oxidation requires hydration, ring-cleavage, rearrangement, cyclization, dehydration, and *O*-methyl cleavage. At least some of these steps are likely to be enzyme-catalyzed.

Recently we embarked on a study to assign functions to the remaining uncharacterized genes in the AF biosynthesis pathway. Among these is *nadA*, which, based on microarray studies, belongs to the AF biosynthesis gene cluster [11] rather than to an adjoining sugar utilization gene cluster as originally proposed [15]. This gene is predicted to encode an OYE (old yellow enzyme) FMN-binding domain (NCBI conserved domain database, cd04733) reductase. While this work was underway, Cai *et al* [3] showed that *nadA* encodes an enzyme that is involved in formation of AFG_1_ and isolated an intermediate with mass 360 Da from *nadA* mutant cultures. They describe this compound as the immediate AFG_1_ precursor [3]. We now provide evidence that *nadA* mutants initially accumulate a 386 Da compound, the probable CypA oxidation product. Additionally, we provide evidence that NorB is responsible for the final oxidation step in AFG_1_ formation.

## 2. Results

### 2.1. Production of gene disruptants

Disruption of *nadA*, *norA* or *norB* was achieved by transformation of *A. parasiticus* BN9Δ*ku70* with the insert portion of plasmid constructs in which the *A. oryzae* pyrithiamine reductase gene, *ptr*, displaced part of the coding region of each gene (see Experimental Section). At least ten colonies were obtained from each transformation experiment. Correct insertion of the disrupted gene by homologous recombination was checked by PCR with either the outer oligonucleotide 5′- and 3′-primers chosen for plasmid construction (*norAK/H* and *norBK/H* oligonucleotides) or with primers internal to the DNA portion used for construct preparation (*nadA*). Formation of a 3.5 kb PCR product compared to a 1.5 kb for the wild-type confirmed that the 2.0 kb *ptr* selection marker was inserted correctly in the *nadA* transformants (Figure 1, lanes 1–5).

*norB* and *norA* disruptants were checked similarly for the correct insertion (Figure 1, lanes 6–7 and 8–9, respectively) and, in these cases, a 3.0 kb band indicated correct insertion, while a 1.2 kb band indicated the intact gene. The frequency of transformants with correctly targeted insertion was higher than 80%.

### 2.2. TLC analyses of extracts of BN9ΔnadA, BN9ΔnorA and BN9ΔnorB

**Figure 2.**
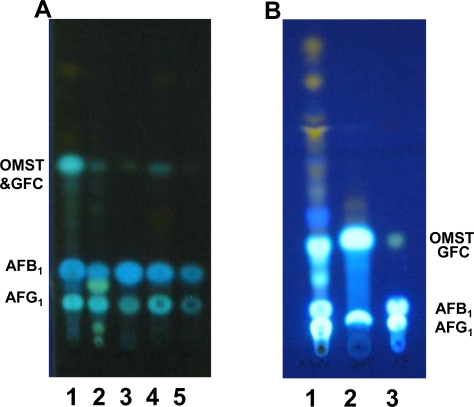
TLC of acetone extracts of *A. parasiticus* BN9Δ*nadA*, BN9Δ*norB*, and BN9Δ*norA.* (A) Lane 1, BN9Δ*nadA* clone 6; lane 2, BN9Δ*norB* (clone B19); lane 3, OMST, AFB_1_ and AFG_1_ standards; lane 4, BN9Δ*norA* extract; lane 5, BN9Δ*ku70* transformed with a plasmid containing *ptr1* (clone B4). (B) Lane 1, unpurified BN9Δ*nadA* extract; lane 2, TLC of purified GFC from BN9Δ*nadA* clone 6; lane 3, standards as above.

A greenish-blue fluorescent compound (GFC) was present in acetone extracts of *nadA* disruptant cultures but not in the parental culture. Upon TLC the GFC almost co-migrated with OMST (Rf 0.38) (Figure 2A, lane 1). When the GFC was completely separated from AFG_1_ by preparative TLC, a small amount of AFG_1_ still appeared in the product mixture (Figure 2B, lane 2). Colonies of BN9Δ*norB* produced several compounds that appeared as greenish-blue fluorescent bands on TLC (Figure 2A, lane 2) that were not found in the untransformed strain (Figure 2A, lane 5). Extracts of cultures of BN9Δ*norA* accumulated slightly elevated amounts of OMST and several reddish-brown compounds compared to the control.

### 2.3. LC/MS analysis of transformant extracts

LC/MS analysis was done on extracts from BN9Δ*nadA*, BN9Δ*norB*, BN9Δ*norA*, and the BN9 control cultures. Based on the relative peak heights in the LC profiles, the ratio of LC fraction peaks with retention times and mass to charge ratios (*m/z*) expected for AFB_1_ (*m/z* 313, 4.5 min) and AFG_1_(*m/z* 329, 4.3 min) was 1.4 for the BN9Δ*nadA* extract, 4.0 for the BN9Δ*norB* extract, 0.8 for the BN9Δ*norA* extract, and 0.9 for the BN9 control strain extract (Table 1).

Metabolites with ion peaks *m/z* 387 and 355 were found in the BN9Δ*nadA* (clone 6) extract (Table 1A) that were not present in the BN9 control extract (Table 1E). In the unpurified BN9Δ*nadA* extract, the compound with the *m/z* 387 ion eluted at 5.3 min in the LC profile and the *m/z* 355 ion eluted at 4.3 min. Under these LC conditions, authentic HOMST (*m/z* 355) eluted at 4.3 min. The Rf 0.38 TLC-purified material from the BN9Δ*nadA* extract contained two compounds with the *m/z* 387 ion peak, one eluting at 4.1 min and the other at 5.4 min (Table 1B). Compounds in the BN9Δ*nadA* TLC-purified material with ion peaks at *m/z* 329 and 339 co-eluted with standards of AFG_1_ and OMST at 4.3 min and 5.0 min, respectively. MS^n^ analysis of the main 387 (5.4 min) ion peak in the BN9Δ*nadA* extract revealed an ion tree with daughter ions at *m/z* 329, 311, 283 and 243. A repeat LC/MS analysis (not shown in Table 1) using a shallower acetonitrile/formic acid gradient instead of a steeper acetonitrile/trifluoroacetic acid gradient revealed the presence of a compound with a prominent ion peak at *m/z* 361 with a retention time of 13.9 min. The *m/z* 387 ion was not found. The retention time of the 360 Da compound was slightly later than AFB_1_ (13.7 min) and the compound had a chromophore in its UV spectrum with λ_max_ = 340 nm.

### 2.4. LC/MS analysis of BN9ΔnorA and BN9ΔnorB transformant extracts

The BN9Δ*norB* (clone 19) extract differed from the control extract in that it had prominent *m/z* 363 and 355 ion peaks at 3.7 min and 4.2 min, respectively. The BN9Δ*norA* extract had an LC/MS profile nearly identical to that of the BN9 control (Table 1D). In agreement with the TLC results, ion peaks at the expected retention times and masses for AFB_1,_ and AFG_1_ were present in all of the unpurified extracts; a peak with the expected retention time and mass for HOMST was detected in the extracts from the BN9Δ*nadA* and BN9Δ*norB* cultures. The repeat LC/MS of the BN9Δ*norB* extract revealed the presence of compounds with prominent ion peaks at *m/z* 303 (12.9 min, λ_max_, 330 nm), 327 (13.9 min, λ_max_, 368 nm), and 359 (14.2 min, λ_max_, 370 nm). Under these LC conditions HOMST, AFB_1,_ AFG_1_, and OMST eluted at 12.4, 12.9, 13.8, and 15.9 min, respectively.

### 2.5. Comparsion of *NadA* in A. parasiticus and A. flavus

**Figure 3.**
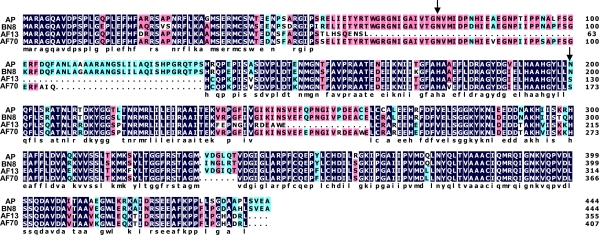
Alignment of the deduced protein sequences for NadA from two B- and G-group of AF-producing species: *A. parasiticus* (AP) and BN008E (BN8), and two sclerotial morphotypes of the B-group of AF-producing *A. flavus*: AF70 (S-strain) and AF13 (L-strain). Asparagine residues (N), presumed important for NadA catalytic function, are marked with vertical arrows.

A 444 amino acid protein and a coding sequence with a single intron are predicted for *nadA* in *A. parasiticus* SU-1 and BN008 isolates based on an analysis using the Softberry FGENESH coding sequence finder software for *Aspergillus* genes. However, the coding sequence for *nadA* from S-strain *A. flavus* AF70 (GenBank accession number AY510453) is predicted to have two introns and to encode a 407 aa protein while the coding sequence for *nadA* in L-strain *A. flavus* AF13 (GenBank accession number AY510451) is predicted to have 5 introns and to encode a 355 aa protein (Figure 3). BLASTp analysis showed that all of the predicted proteins possess a conserved OYE-FMN reductase family domain. In the deduced sequences of the *A. flavus* proteins, a glutamine that may be necessary for catalytic activity is missing [2]. The predicted AF13 NadA sequence differs substantially in other regions as well.

## 3. Discussion

*A. parasiticus nadA* disruptants accumulate a 386 Da greenish-blue-fluorescent (GFC) metabolite that is not found in strains with the intact gene. A compound with this molecular weight is consistent with the predicted lactone (**3**, Scheme 1) or one or more of its isomeric forms (not shown) created by sequential addition to HOMST (354 Da) of two oxygen atoms by the cytochrome P450 monooxygenases, OrdA and CypA (Scheme 1) [7, 13]. This is the first report providing experimental evidence for the presence of this compound in *A. parasiticus* extracts. The TLC and LC/MS results suggest that this compound readily loses CO_2_ non-enzymatically and rearranges to AFG_1_. The ion tree of the 386 Da compound revealed by MS^n^ analysis indicates that this compound fragments with loss of a *m/z* 58 ion (CO_2_ and CH_2_) or a *m/z* 72 ion (CO_2_ and CH_3_OH), a pattern consistent with its identification as an AFG_1_ precursor.

In addition to the 386 Da compound, AFB_1_, AFG_1_, HOMST and OMST (Table 1) accumulate in the *nadA* knockout culture as determined by comparison of retention times and UV chromophores to those of control samples. This is the first report that HOMST, a proven substrate for OrdA-catalyzed conversion to AFB_1_ [13], is present in metabolite extracts of *A. parasiticus* cultures. The extracts from the *A. parasiticus* BN9 control and BN9Δ*nadA* and BN9Δ*norB* cultures also contain a compound with a prominent *m/z* 371 ion. Based on its mass and LC retention time relative to that of AFB_1_ this compound is most likely the predicted oxidation product of OMST [13] and the precursor **2** of both AFB_1_ and AFG_1_ as shown in Scheme 1. Although norsolorinic acid, an early metabolite in AF biosynthesis, also has a positive ion MS peak at *m/z* 371, it has a markedly different retention time under these conditions on LC (7.3 min for norsolorinic acid vs. 3.7 min for the 370 Da metabolite).

Sequence analyses indicate that *A. parasiticus* NadA has a conserved domain (cd04733; http://www.ncbi.nlm.nih.gov/Structure/cdd/cddsrv.cgi?uid=cd04733) characteristic of old yellow enzyme (OYE)-related FMN binding domain reductases [14]. Proteins with such domains are predicted to use NADPH to reduce the carbon-carbon double bond of α,β-unsaturated-aldehydes or ketones [8]. *A. parasiticus* NadA was found to contain amino acids predicted for such reductases. These include amino acids His-195 and Asn-200 involved in binding the oxygen atom of the substrate, and Tyr-197 required for proton transfer to the β-carbon atom of the α,β-unsaturated ketone to complete the reduction initiated by hydride transfer from the N-5 atom of flavin mononucleotide.

Based on predicted intron assignments for *nadA* from the two types of *A. flavus*, the proteins for NadA would differ considerably from those predicted for *A. parasiticus* and an isolate of the *A. parasiticus*-like species, BN008 [15] (Figure 3). Such differences are unexpected since the sequences and intron assignments of the other AF biosynthesis genes are the same among the different AF-producing species [15]. Furthermore, the conserved Asn-76 thought to be involved in flavin-binding is missing and the catalytic Asn-200 is replaced by a Ser in *A. flavus* NadA. These amino acid changes are expected to decrease or prevent enzyme activity [8]. Transcripts of *nadA* have been identified in *A. flavus*, but no studies have been done to show if a functional enzyme is encoded by these transcripts. The predicted sequence of *A. flavus* AF13 NadA also is missing other portions of the coding sequence that also may contain critical amino acid residues (Figure 3). Based on these sequence differences, we predict that NadA from *A. flavus* AF13 (L strain) and possibly from AF70 (S strain) is not functional. To form AFB_1_ in *A. flavus* a reduction step may also be involved (Ehrlich *et al* unpublished results), but NadA is unlikely to play a role. Another reducing agent, possibly Nor-1 the reductase involved in conversion of norsolorinic acid to averantin in the first AF bioconversion step, may be involved in formation of AFB_1_ in *A. flavus.*

**Scheme 2.**
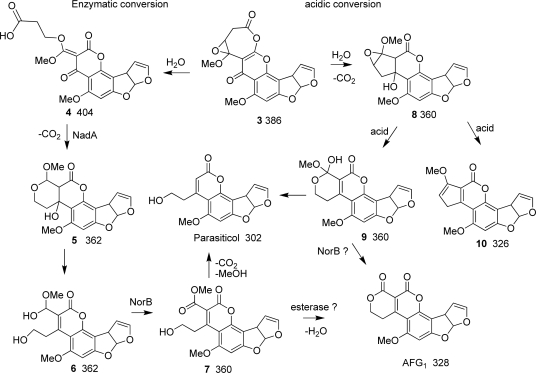
Scheme showing some possible intermediates in the conversion of the 386 Da lactone **3** to AFG_1_. NadA-catalyzed reduction is assumed to follow lactone hydration and ring-opening (see Discussion). The quinone (**4**) resulting from ring-opening of the 386 Da intermediate is shown as the substrate for NadA. The 362 Da alcohol (**6**) is shown as the substrate for NorB. Non-enzymatic conversion of the 386 Da lactone to AFG_1_ is also shown.

A 360 Da intermediate (**7**, **8** or **9** in Scheme 2) was obtained from a repeat analysis of a BN9Δ*nadA* extract. This compound may be the same as that partially characterized by Cai *et al.* [6] in a similar *nadA* knockout culture from *A. parasiticus*. However, because NadA is an OYE FMN-domain reductase, it is unlikely to catalyze the oxidation of the 360 Da intermediate to AFG_1_. A necessary step in the conversion of **8** to the alcohol **9** involves epoxide ring-opening. NadA-catalyzed reduction may facilitate the subsequent epoxide ring-opening and rearrangement of the vinylogous ester **4** shown in Scheme 2. In the absence of NadA, ring-opening and rearrangement may occur only when the 386 Da metabolite is exposed to the acidic conditions of the culture medium during the extraction process, a possibility that could explain the apparent ability of *nadA* mutants to form AFG_1_ in the absence of enzyme.

*A. parasiticus* BN9Δ*norB* cultures accumulate 362 and 326 Da yellowish-green fluorescent metabolites in addition to HOMST, OMST and AFs, but they do not accumulate the 386 Da metabolite. The presence of these metabolites is consistent with the proposition that NorB catalyzes an oxidation step after NadA reduction. In the scheme in Scheme 2, the 362 Da rearrangement product (**5** or **6**) may be oxidized by NorB to produce an ester (**7**), which could be enzymatically de-esterified by numerous fungal esterases. This product upon dehydration would yield AFG_1_. Oxidation of the alcohol **6** to form **7** is consistent with the presumed enzymatic capability of NorB. Although both *norA* and *norB* are predicted to encode similar NAD^+^ or NADP^+^-dependent alcohol dehydrogenases, *A. parasiticus* transformants lacking functional NorA showed essentially no change in their abilities to produce AFs compared to a control culture. Therefore, NorA presumably is unable to completely complement the activity of NorB. In the absence of NorB the epoxide ring could undergo an alternative acid-catalyzed ring-opening to give a diol that, upon dehydration, gives the methyl ether of AFB_1_ **10**.

Based on its predicted activity and its involvement after NadA in AFG_1_ formation, we suggest that NorB catalyzes the biosynthesis step after the rearrangement and decarboxylation of the NadA-reduced 386 Da intermediate. Upon repeat LC/MS three additional compounds with M+H ions at *m/z* 303, 327, and 359 were detected that were not found in the control culture extract. The chromophores of these compounds are consistent with derailment products resulting from non-enzymatic breakdown of one or more of the precursor metabolites upon prolonged storage. The 303 ion is most likely to be parasiticol (302 Da), a compound often found in aged cultures of extracts containing AFG_1_ [12]. Parasiticol could be a degradation product of the alcohol **7** after losses of CO_2_ and MeOH. The *m/z* 327 ion indicates a compound resulting from net loss of an oxygen atom and CO_2_ from the 386 Da lactone **3** or an oxygen atom and water from either of the 360 Da intermediates, **7** or **8**. The *m/z* 359 in the repeat LC/MS analysis suggests the net loss of a carbonyl from the 386 Da lactone **8**. Such chemical changes are consistent with the presumed instability of the AFG_1_ intermediate(s) in the presence of acid and the chromophores detected for these compounds.

Acid-catalyzed hydrolysis and decarboxylation of the 386 Da intermediate **3** could give an unstable 360 Da compound **8** or **9** which may spontaneously rearrange to AFG_1_ (Scheme 2). Because extracts of both the *nadA* and *norA* knockout mutants still contain AFG_1_, an acid-catalyzed conversion process probably is allowed to occur when the cells are lysed in an acidic environment. The ester **7** resulting from ring-opening of **9** or NorB oxidation of **6** may be an intermediate in the conversion of **3** to AFG_1_. Parasiticol and the 326 Da AFB_1_ methyl ether could be degradation products of this intermediate ester.

## 4. Experimental Section

### 4.1. Construction of gene knockout vectors

PCR and standard recombinant DNA techniques were used to prepare plasmids for insertional inactivation of the *Aspergillus* genes *ku70*, *nadA, norA,* and *norB* in *A. parasiticus* isolate BN009E ([7], BN9). To increase gene-targeting frequency in fungal transformants, a *ku70*-mutant was created in *A. parasiticus* BN9*niaD*^−^. Disruption of *ku70* allows selection of a higher frequency of homologous recombination. Preparation of the *ku70* mutant involved construction of a disruption plasmid, PCR amplification and sequential cloning into pUC19 of a 1.4-kb 5′-untranslated region (UTR) and a 1.7-kb 3′-UTR using the oligonucleotide primer pairs ku5Sm: CTTCGCCCGGGTACGGGTCACCT AATC and ku5X: TATCTAGAGTCGTAAGTCATGAATTGCGT and ku3X, ATTCTAGACAACG CTAGTATTGGTTACGAG and ku3S, CTAGAACGAATTCGTGTCGACACTGA. Following this, a 6.7-kb *Xba*I fragment that contained *A. parasiticus niaD* selectable marker [6] was inserted into the plasmid to give pA *ku70*. Prior to transformation the pA *ku70* was digested by *Sma*I and *Sal*I and Δ*ku70* transformants selected. Protoplasting, transformation and selection were done according to a previously described procedure [5].

The resulting BN9Δ*ku70* was the recipient strain for subsequent fungal transformations. The *nadA* disruption vector was prepared by PCR using the following primers: nad5KH, ATCAGGTACCGACTGCCCCCAAGCTTCAAGC; nad5X, ATCCGGCTCGATCTAGAAACTCG, nad3X, GTTTGATTCTAGAGATCCGTGC; nad3H, GAAGGCTAAGCTTCGACTGATAAG (nt 70029 to 70981 and 71125 to 71825, respectively in GenBank Acc. #AY371490) to generate DNA fragments that included portions of the *nadA* 5′-promoter and coding region and the 3′-coding region. The PCR fragments were cloned into pUC19 followed by insertion of the *A. oryzae* pyrithiamine resistance gene (*ptr*) [10] into the filled-in *Xba*I site. The *ptr* fragment was amplified from pPTR1 (Takara, Japan) with primers ptr730P: ATACTGCAGACGGGCAATTGATTACGG and ptr1230P: TTACTGCAGCCGCTCTTGCATCTTTG. The resulting vector was linearized with *Hin*dIII prior to transformation into BN9Δ*ku70*. To confirm the disruption of the *nadA* gene, PCR of DNA from single-spore cloned transformants was done with primers, nad1500, 5′-TATGCTTCCTTTGTACCGATCA and nad2970, ATTGCCGTGCACCAGAGATC (nt 70397 and 71882 in AY371490).

Similarly, 0.4-kb and 0.6-kb portions of the *norB* 3′-coding and UTR and 5′-*norB* coding region were generated by PCR with primers norBH, GCAAAAGCTTCAGACGAGCTAT and norBF, TGTCCAACACTCTGCAGACGCTG, and norBK: CGTTGGAAACAGGTACCAATGG and norBR: CACTCTGCAGACGCTGCGATCAT (nt 1427 to 2424 and 506 to 2041 in AY371490). The PCR fragments were cloned into pUC19 followed by the insertion of the above *ptr* fragment into the *Pst*I site. The resulting vector was linearized by *Hin*dIII and *Kpn*I prior to transformation of BN9Δ*ku70*. Primers norBH and norBK were used in PCR to confirm *norB* gene disruption. A *norA* disruption vector was similarly prepared using the primers norAH, 5′-CGATAAGCTTGTAAGGCATTCT and norAK 5′-CTCGGTACCAAGCCGAGAGCCT (nt 39851 to 40911 in AY371490) for PCR. The *ptr* fragment was inserted into a *Pst*I site 560 bp from the 5′-end. Fungal transformation and selection of pyrithiamine resistant colonies was done as previously described [5] or as described in the product manual for *ptr* selection by Takara-Bio, Inc (http://www.takara-bio.com/research.htm).

### 4.2. Thin layer chromatography (TLC) and liquid chromatography mass spectrometry (LC/MS)

Dried samples of the chloroform/acetone extract of liquid cultures grown at 22 °C on potato dextrose broth (PDB) medium were analyzed by TLC on 250 μm silica gel plates (J.T. Baker), developed with toluene-ethyl acetate-acetic acid (8:1:1) or by LC/MS on a 3x50 mm Betasil (Thermo Fisher Scientific) column using either a 7 to 10 min, 10% acetonitrile: 0.025% trifluoroacetic acid to 95% acetonitrile: 0.025% trifluoroacetic acid gradient (HT Laboratories, San Diego CA); http://www.ht-labs.com;) or on a Luna C18 100 × 4.6 mm column (5 μm, 100Å, Phenomenex) using a 30 min 10% acetonitrile/0.1% formic acid and 90% water/0.1% formic acid to 99.9% acetonitrile/0.1% formic acid gradient. LC detection was by photodiode array at 215 nm and MS was done using atmospheric pressure chemical ionization. Metabolites were monitored by both diode array UV-visible spectrophotometry and quadrupole MS (Agilent 6130). MS^n^ analysis (mass spectrometry performed in sequential stages) was run until signal detection was no longer possible.

### 4.3. Sequence comparison

Sequences of the *nadA* regions from *A. parasiticus* SU-1 (AY371490), *A. flavus* AF13 (GenBank accession number AY510451), an isolate of the unnamed B and G-accumulating *Aspergillus* taxon (BN008) (AY510453), were aligned by DNAMAN. Assessment of coding regions was done using FGENESH (Softberry, http://www.softberry.com/berry.phtml) software. BLASTp searches were done against the non-redundant protein database (http://www.ncbi.nlm.nih.gov/sites/entrez?db=pubmed).

## 5. Conclusions

The genes *nadA* and *norA* encode enzymes involved in AFG_1_ formation. In the absence of *nadA* a 386 Da intermediate accumulates that is the product expected to result from OrdA and CypA oxidation. In the absence of *norB*, 360 and 362 Da compounds accumulate that are immediate precursors of AFG_1_. Other products accumulating in the transformants, namely parasiticol and a 326 Da compound are likely to be derailment products of these intermediates.

## Figures and Tables

**Table 1.** LC/MS data from *A. parasiticus* BN9Δ*nadA*, BN9Δ*norB*, and BN9Δ*norA* extracts The mass spectrometry was in positive ion mode. Retention times on LC are normalized to the retention time of AFB_1_ to account for slight variations in different LC runs. Retention times in min of standards were: AFB_1_, 4.5; AFG_1_, 4.3; HOMST, 4.2; and OMST, 5.0. The mass to charge ratios (*m/z*) are rounded off to the nearest whole number. Only odd ion peaks and peaks less that *m/z* 400 are listed. The relative peak intensity is the percent peak height relative to the highest peak in the mass spectrum.

LC Peak	Retention time, min (relative peak height)	Mass/charge [M+H^+^] (relative peak intensity)	
**A. BN9Δ*nadA***		**Peak 1**	**Peak 2**
1	3.7 (4)	371 (100)	363 (6)
2	4.3 (69)	329 (100)	355 (15)
3	4.5 (100)	313 (100)	329 (39)
4	5.3 (2)	387 (100)	339 (24)
**B. BN9Δ*nadA*-TLC**			
1	4.1 (50)	329 (100)	387 (15)
2	4.3 (100)	329 (100)	383 (7)
3	5.0 (7)	339 (100)	291 (46)
4	5.4 (20)	387 (100)	
**C. BN9Δ*norB***			
1	3.7 (23)	363 (100)	361 (18)
2	4.2 (5)	355 (100)	371 (6)
3	4.3 (23)	329 (100)	311 (7)
4	4.5 (100)	313 (100)	327 (37)
5	5.0 (3)	339 (100)	
**D. BN9Δ*norA***			
3	4.3 (100)	329 (100)	311 (9)
4	4.4 (82)	313 (100)	329 (25)
5	5.0 (20)	339 (100)	295 (5)
**E. BN9control**			
1	4.3 (100)	329 (100)	311 (6)
2	4.5 (94)	313 (100)	285 (5)
3	5.0 (16)	339 (100)	295 (2)
